# Embracing Diversity in Mental Health Education: A Primary Study on Cultivating Cultural Humility in Undergraduate Medicine

**DOI:** 10.1192/bjo.2024.280

**Published:** 2024-08-01

**Authors:** Ariana Axiaq

**Affiliations:** Queen's University Belfast, Belfast, United Kingdom

## Abstract

**Aims:**

Training within medical schools often neglects that mental health patients are very culturally diverse, contributing to a lack of cultural competence in future doctors. This deficiency exacerbates access to healthcare barriers for this population. To address these issues early in the course, we initiated a student-led teaching programme on Cultural Humility (CH) for first-year medical students, aiming to enhance cultural awareness (CA) about mental health patients.

CH emphasises the lifelong development of skills, knowledge, and attitudes, fostering a perspective of ‘becoming the student of the patient' to address power imbalances between doctors and patients with mental illness, particularly from minority groups. It promotes culture as expansile incorporating many characteristics from race and religion to sexual orientation, disability and age.

This study assessed the knowledge and perceptions of first-year medical students following the introduction of CH.

**Methods:**

After exposure to an author-developed CH learning resource, students participated in a baseline survey to gauge their understanding of CH. Subsequently, an interactive student-led workshop with a reflective exercise encouraged medical students to embrace their cultural diversity and that of others, emphasising the multifactorial nature of mental illness. The workshop incorporated prompts inspired by peer experiences of mental illness. Students then engaged in an early clinical contact programme, interacting with patients with mental illness to implement their understanding of CH into practice. Reflective blogs, written by students as part of the programme, were analysed for data inclusion using an author-selected framework.

**Results:**

Out of 312 participants, 188 provided responses, revealing higher scores for perceived CH importance (4.83/5) compared with understanding (3.86/5) and perceived preparedness for CH implementation (3.98/5). Analysis of free-text survey responses identified learning gaps, particularly in demonstrating cultural sensitivity during patient interviews, and highlighted preferred pedagogies. Thematic analysis of ten collected blogs followed a 5R framework: Respect, Reflection, Regard, Relevance, and Resiliency. Findings indicated a demand for better training in identifying patient-specific sensitive topics and a preference for appreciating patient characteristics such as socioeconomic class without explicit labelling of these qualities as culturally engendered or directly linked to their diagnosis.

**Conclusion:**

CH aims to foster a patient-centred approach, encouraging medical students to look beyond the diagnosis of mental illness. This study explored the multimodal integration of CH as a CA toolkit into the undergraduate curriculum, providing insights into the application of preclinical CA teaching and students’ perceptions of its clinical applicability in their learning about different patient populations experiencing mental illness.

